# Individual variation in parental workload and breeding productivity in female European starlings: is the effort worth it?

**DOI:** 10.1002/ece3.1625

**Published:** 2015-08-06

**Authors:** Melinda A Fowler, Tony D Williams

**Affiliations:** 1 Department of Biological Sciences, Simon Fraser University8888 University Dr, Burnaby, BC, V5A 1S6, Canada

**Keywords:** Female parental care, individual variation, provisioning, reproductive success, workload

## Abstract

We analyzed individual variation in work load (nest visit rate) during chick-rearing, and the consequences of this variation in terms of breeding productivity, in a highly synchronous breeder, the European starling (*Sturnus vulgaris*) focusing on female birds. There was marked (10- to 16-fold) variation in total, female and male nest visit rates, among individuals, but individual variation in female nest visit rate was independent of environment (rainfall, temperature) and metrics of individual quality (laying date, clutch size, amount of male provisioning help), and was only weakly associated with chick demand (i.e., day 6 brood size). Female nest visit rate was independent of date and experimentally delayed birds provisioned at the same rate as peak-nesting birds; supporting a lack of effect of date per se. Brood size at fledging was positively but weakly related to total nest visit rate (male + female), with >fivefold variation in nest visit rate for any given brood size, and in females brood size at fledging and chick mass at fledging were independent of female nest visit rate, that is, individual variation in workload was not associated with higher productivity. Nevertheless, nest visit rate in females was repeatable among consecutive days (6–8 posthatching), and between peak (first) and second broods, but not among years. Our data suggest that individual females behave as if committed to a certain level of parental care at the outset of their annual breeding attempt, but this varies among years, that is, behavior is not fixed throughout an individual's life but represents an annually variable decision. We suggest females are making predictable decisions about their workload during provisioning that maximizes their overall fitness based on an integration of information on their current environment (although these cues currently remain unidentified).

## Introduction

Individual-based resource allocation trade-offs associated with costs of reproduction represent central themes of life-history theory, and one of the most widely accepted sources of such costs are those associated with reproductive investment in rearing offspring, that is, parental care (Stearns 1989; Clutton-Brock 1991; Royle et al. 2012; Williams 2012b). In particular, life-history theory predicts a) that individuals that invest more in parental care should benefit in terms of rearing an increased number of offspring, or larger, fitter offspring, but that b) increased investment in parental care might come at a cost in terms of decreased future fecundity and/or survival (Schroeder et al. 2013). Surprisingly, there is equivocal evidence to support either of these predictions in birds, especially for females (reviewed in (Mitchell et al. 2012; Santos and Nakagawa 2012). In particular, the idea that parents that work harder, e.g., provisioning chicks at a higher rate, produce more fitter chicks is surprisingly poorly supported (Schwagmeyer and Mock 2008; Williams 2012b) see below). The number of chicks fledged (McCleery et al. 2004) or the mass or size of those chicks is considered an important component of fitness: Fledging mass has been shown to be an important predictor of postfledging survival in many studies (e.g., Schwagmeyer and Mock 2008; Cleasby et al. 2010; Mitchell et al. 2011; Dybala et al. 2013). However, while a number of studies have reported positive relationships between parental care (nest visit rate) and the current number of chicks (Shutler et al. 2006; Ardia 2007; Bortolotti et al. 2011; Garcia-Navas and Sanz 2011), the relationship between nest visit rate and reproductive success at fledging is less clear; in many studies, chick mass and brood size at fledging are independent of marked interindividual variation in adult nest visit rate (Dawson and Bortolotti 2003; Schwagmeyer and Mock 2008; Ringsby et al. 2009; Mariette et al. 2011; Garcia-Navas et al. 2012; Williams 2012a). Why would individuals sustain high nest visit rates, and risk incurring potential costs of this high reproductive effort, if there is no clear benefit of this higher workload?

The inconsistent patterns between nest visit rate and resulting offspring quality (see above) could be due to individual differences among provisioning parents in the relative costs and benefits of particular levels of workload. Although birds could vary workload through variation in meal size, foraging distance, or the size or quality of prey (Wright et al. 1998; Stodola et al. 2010; see Discussion), individual variation in reproductive effort is often explained using the “date” versus “quality” paradigm (Verhulst and Nilsson 2008). The date hypothesis assumes that the benefits (and costs) of parental expenditure are time dependent because the reproductive value of offspring declines seasonally (Clutton-Brock 1991; Drent 2006). The hypothesis assumes either a) time per se is important, for example, later fledged offspring have less time to prepare for molt or migration, with lower survival or b) because environmental quality declines seasonally (e.g., decreased food, increased parasites; Dzus and Clark 1998; Brown and Brown 1999), making it harder to rear good quality offspring with higher survival probabilities. In contrast, the quality hypothesis suggests that there are inherent differences among individuals in their phenotypic quality, such that brood size and chick quality might differ between individual pairs depending on their competence in raising young (Drent 2006), independently of date or environmental conditions (although it is possible that “date” and “quality” can interact). Phenotypic quality is a widely used but poorly defined term (Wilson and Nussey 2010), but in birds, higher quality individuals generally lay larger clutches with earlier laying dates (Williams 2012b), have higher foraging efficiency (Daunt et al. 2006; Lescroël et al. 2010), and higher breeding success (Hamel et al. 2009; Wilson and Nussey 2010). In addition, in avian species with bi-parental care, mate choice may also be a signal of quality (Schwagmeyer and Mock 2003), for example, females that can attract high-quality mates that provision more might then produce more lifetime recruits (Schuett et al. 2010; Schroeder et al. 2013). In support of the quality hypothesis, individuals where timing of reproduction is experimentally delayed are expected to perform at the same level as higher quality “peak” nesting birds (i.e., first broods), despite raising chicks at a later date. Alternatively, if delayed birds have different provisioning behavior or reproductive success from peak birds that reared chicks earlier, this provides support for an environmental or date effect.

Here, we use a 10-year data set to analyze sources of variation in work load (nest visit rate) during chick-rearing in European starlings (*Sturnus vulgaris*) and the consequences of this variation in terms of breeding productivity, focusing on individual variation in *female* birds. Specifically, we firstly quantify variation between individuals in total, male and female nest visit rates, within- and across years, and show that environmental variation (temperature and rainfall) does not contribute to variation in nest visit rates (i.e., these do not confound our subsequent analyses). We then address within-individual variation by (1) estimating repeatability of individual nest visit rates within years (between first and second broods) and among years. We test the predictions that, (2) between individuals, higher nest visit rates are associated with metrics of individual female quality (e.g., laying date, clutch size, amount of male provisioning help) and (3) that pairs or females with higher nest visit rates fledge larger broods with larger mean chick fledging mass. Secondly, we explicitly test the “date hypothesis” to explain between individual variation in nest visit rate in the context of the high breeding synchrony of European starlings (80% of nests being initiated over a 4.8 ± 1.4 day period, range 2–8 days, *n* = 944 nests, 13 years; (Williams et al. 2015). This high degree of breeding synchrony provides a natural experiment where potential date effects (i.e., changes in environment) are naturally controlled for in the analysis of individual variation in quality (above), but in some years, we also experimentally delayed timing of breeding in high-quality, peak-nesting females (by removing their first clutch, e.g., Love and Williams 2008) to investigate the role of date versus quality by comparing nest visit rate among experimentally delayed replacement clutches, naturally late laying birds and peak (i.e., early) broods.

## Methods

### Breeding data

We used 10 years of breeding data (2002–2005, 2007, 2009–2013) from our long-term European starling study at Davidstead Farm, Langley, British Columbia, Canada (49°10′N, 122°50′W), which comprises about 150 nest boxes mounted on posts around pastures and on farm buildings. Each year, we followed the same basic field protocol: Nest boxes were checked daily from April 1 to determine laying date and clutch size. Clutch size refers to the eggs laid, whereas we use brood to refer to how many chicks are alive in the nest. In several years, we conducted experiments which involved catching females at clutch completion and/or removing eggs to stimulate laying of replacement clutches (e.g., Love and Williams 2008), which experimentally delayed chick-rearing. Nests were checked on day 6 and again on day 17 (shortly before fledging) to obtain brood size at fledging (BSF), and chicks were weighed on day 17 to determine mean fledging mass per brood. In each year, individual females were captured during incubation and fitted with color bands and individually numbered metal bands (Environment Canada permit # 10646). Males were not captured or banded, and thus, identity for males is unknown (thus, we focus our analyses on females; see below). All research was conducted under Simon Fraser University Animal Care permits # 657B-96, 829B-96, 1018B-96).

We designated nests as “peak” broods if they initiated laying within a 12-day period from the earliest first nest initiation date in each year (Williams et al. 2015). Pairs that successfully fledged a peak (first) brood often initiated a second breeding attempt. Nests were categorized as true “second broods” if they were initiated within a period determined by the earliest laying date of birds known to have successfully fledged a peak brood. Nests initiated between the peak and second broods were categorized as intermediate, including either “experimentally delayed” broods (birds that first laid in the peak window, but where eggs were removed to stimulate laying at a later time) or “natural” intermediate broods.

### Provisioning data

Provisioning observations to record nest visit rate were conducted between 0900 and 1400 on days 6–8 posthatching (day 0 was defined as the day the majority of chicks in the nest hatched). Nest visit rate for each nest was based on 30-min observations with binoculars or a spotting scope (units: nest visits/30 min). Brooding at this stage is seldom observed (Tinbergen 1981), but if birds remained in the nest box for >1 min during observations, this was noted and deducted from the observation period for calculation of nest visit rate. Most observations were performed from inside a vehicle (a mobile blind), to which the birds are acclimated (the site is on a farm and near several houses and a roadway). Where this was not possible a spotting scope was used from a greater distance. If the observer was detected (birds under observation would alarm call), the observation was ended, the data discarded, the observer moved farther away, and the observation restarted. Overall, 73% of the observations were repeated 3 times during the 6–8 day period and 27% were performed twice. To assess the robustness of our provisioning measures, we used a resampling technique. For birds with three provisioning observations, we used the random selection function in Excel to randomly select from the three points 250 separate times. We then took the average of two of these randomly selected points for each individual, resulting in 125 values for each individual. We used R to calculate the correlation between all the three-point-averaged values and the randomly selected two-point means, resulting in a correlation coefficient. We did this for all 125 of the means calculated from the random selection of two points. We then average all 125 correlation coefficients to arrive at the number reported. Randomly resampling two of three data points for total nest visit rate correlated very tightly with the average of the three data points (mean *r* = 0.90, SEM = 0.006), giving us a high degree of confidence that our behavioral metrics were consistent between days. Similarly, resampling two of three data points for female provisioning rate correlated very highly with the average of the three data points (mean *r *=* *0.78, SEM = 0.02). Nest visit rate data were therefore averaged over the multiple days of observation. During the 30 min observation period, nest visits from females and males were counted, based on the presence of color bands on the female (males have no color bands). Visits were categorized as unknown if the leg of the visiting bird was not seen clearly and unknown visits were partitioned between males and females based on the ratio of known-sex visits. Thus, although we did not know the individual identity of males, or the females genetic mate, we could measure nest visit rate of the female's social partner, that is, the male contributing to feeding of a female's offspring. So while we could not address the issue of polygyny (Sandell et al. 1996) directly, we could address the consequences of polygyny from the female perspective: Was breeding productivity (brood size at fledging, chick fledging mass) higher for females with male help vs. no male help.

### Temperature data and analysis

Daily temperature data were obtained for the Pitt Meadows weather station, British Columbia (49°12′N, 122°41′W, elevation 5.0 m asl), using the Environment Canada online National Climate Data and Information Archive (http://www.climate.weatheroffice.gc.ca). Pitt Meadows is <20 km from both our study sites at Davidstead Farm, Langley (49°10′N, 122°50′W), and Glen Valley, Langley (49°10′N, 122°28′W). Mean monthly temperature at Pitt Meadows was highly correlated (*r* ≥ 0.95, *P* < 0.001, originally calculated in SAS) with mean monthly temperature at the Cloverdale weather station (20 km south-east of our study sites), and at Vancouver Airport (40 km west), and thus provides a good index of variation in regional temperature (see Williams et al. 2015). We calculated mean daily temperature for the 3 days for each individual nest's provisioning observation period. Likewise, daily rainfall for the 3 days of observations was averaged.

### Statistical analyses

Analyses were carried out using R version 3.0.1. We used the lme4 package (Bates et al. 2013) with individual female ID and year as random effects (unless noted otherwise below). F statistics and *P* values were generated using df with the Kenward–Rogers correction and the lmerTest package (Kuznetsova et al. 2013). Tukey's HSD (package multcomp, (Hothorn et al. 2008)) was used to evaluate pairwise comparisons between stages following a significant mixed model. An *R*^2^ for mixed models was calculated for significant fixed effects (Edwards et al. 2008). When brood size was investigated as a response variable, we used a generalized mixed effects models with Poisson error distributions, a logarithmic link function and included individual identity and year as random effects. We report the *z*-statistic and associated *P* value. Brood size at day 6 was included as a covariate in models assessing differences in nest visit rate. A summary of the models run, with each response variable, covariates and random effects is shown in appendix Table4.

#### Individual variation in nest visit rate and environmental factors

We ran an exploratory linear mixed model analysis of the environmental variables rain and temperature (averaged over 3 days of provisioning) and their effect on nest visit rate. We found no significant effect of rain and temperature on nest visit rate (total, female or male; see Results) and thus did not include these variables as covariates in further models. Our next step was to assess annual differences between peak broods for nest visit rate and breeding productivity (brood size at fledging and fledge mass). Year was assessed as a fixed effect for this analysis, and thus only individual ID was included as a random effect. Brood size at day 6 was included as a covariate for annual differences total, female and male nest visit rates.

#### Repeatability of nest visit rate and relationship with individual quality metrics

Repeatability (*r*_r_) is a metric often used in behavioral studies to estimate the portion of total variation that is attributed to among individual differences (Lessells and Boag 1987; Bell et al. 2009; Matson et al. 2012). Repeatability was assessed in two ways; both as a linear mixed effect model in the lmer package and with the package rptR (Schielzeth and Nakagawa 2013). When assessing repeatability with mixed effects modeling, we included individual identity and year as random effects. We extracted the variance within and among groups and calculated repeatability as *r*_r_ = variance_among_/(variance_among_ + variance_within_) after Nakagawa and Schielzeth 2010. The variance_among_ included variance components for both random effects, while the variance_within_ included the residual variance. The variance for year was zero in all cases, so we also computed repeatability in the rptR package, which will not compute multiple random effects. We used the rptR.remlLMM function, which also uses the linear mixed effects methodology and returns a *P* value. We included individual bird as the random effect. We report repeatability estimates calculated with both methods, and the permutated *P* value from the rptR package. We use the variable “per chick” nest visit rate in the repeatability analysis only, a very commonly used metric, and per chick nest visit rate was calculated by dividing the provisioning rate by the brood size at day 6. Within peak broods, we tested whether nest visit rate varied with clutch size or lay date (female ID and year included as random effects and brood size at day 6 included as covariate in lay date analysis). Additionally, we modeled total, male and female nest visit rate (in peak broods), as a function of brood size at day 6 with individual bird identity and year included as random effects. We tested whether total female nest visit rate varied in relation to whether males provided any help (nest visit rate > 0) or none at all (male nest visit rate = 0), year and female identity as random effects.

#### Variation in nest visit rate and breeding productivity in peak broods

For fitness metrics, we modeled brood size at fledge (17 days posthatching) as a function of nest visit rate, with generalized mixed effects models as above and individual female bird identity and year included as random effects. Similarly, fledge mass as a function of nest visit rate. Individual female bird identity and year were included as random effects. We also tested whether total, female or male provisioning, brood size at fledge or fledge mass varied between peak, intermediate and second broods. Female ID and year were included as random effects, and brood size at day 6 was included as a covariate in models assessing provisioning as a response variable. To assess the potential effects of polygyny, we tested whether brood size at fledge or fledge mass varied with the presence or absence of male help (male nest visit rate >0 or = 0), year and female ID as random effects.

#### Effect of experimentally delayed timing of chick-rearing on nest visit rate

We tested whether nest visit rate or productivity varied as a function of treatment or just as “date” alone, comparing natural peak broods to experimentally delayed birds. For assessing effect of date in experimentally delayed birds, we tested whether peak broods differed from experimentally delayed birds regarding the following variables: total or female nest visit rate, day 6 brood size, brood size at fledge, and fledge mass. Each of these variables was assessed as the response variable to the treatment variable (“delayed” or not; *n* = 148). We investigated the same response variables as a function of Julian date as well. Female ID and year were included as random effects and when nest visit rates were the response variable, brood size at day 6 was included as a covariate. Finally, we also investigated differences in these traits within the range of intermediate broods, including birds which naturally laid in the intermediate date range (i.e., natural replacement clutches) and the birds that were experimentally delayed (*n* = 54). To assess the difference between natural intermediate broods and experimentally delayed birds, we modeled the following response variables as a function of treatment (“delayed” or not): total and female nest visit rate, brood size day 6, brood size at fledging, fledge mass. Female ID and year were included as random effects and when nest visit rates were the response variable, brood size at day 6 was included as a covariate.

## Results

### Individual variation in nest visit rate and environmental factors

There was marked individual variation in total nest visit rate (both sexes combined) for peak broods (range 1–16 visits/30 min), female nest visit rate (range 0–10 visits/30 min), and male nest visit rate (0–9.9 visits/30 min; Table1). However, within-pairs male and female nest visit rate were not correlated (Pearson's correlation, *r *=* *−0.13). Variation in both total and female nest visit rate was independent of 3 day average rainfall and 3 day average temperature during the provisioning period (*P* > 0.5 in all cases). Furthermore, there were no interannual differences in mean total, female, or male nest visit rate (brood size at day 6 included as a covariate), of peak broods or in brood size at fledging (*P* > 0.05 in all cases; Table2; day 6 brood size was correlated with both clutch size (*r* = 0.59, *P* < 0.001) and brood size at fledge (*r* = 0.89, *P* < 0.001)).

**Table 1:** Mean reproductive success and nest visit rates in European starling peak, intermediate and second broods over 10 years

		Peak	Intermediate	Second
Brood size at fledge	Mean	3.91^a^	3.81^a^	2.83^b^
	SEM	0.11	0.17	0.17
	Range	2–6	1–6	1–5
Total nest visit rate	Mean	6.96^a^	5.75^b^	4.95^b^
	SEM	0.30	0.38	0.36
	Range	1–16.33	1–14	0–12
Female nest visit rate	Mean	4.38	3.94	3.25
	SEM	0.22	0.30	0.26
	Range	0–10	0–11	0–8.9
Male nest visit rate	Mean	2.54	1.79	1.63
	SEM	0.23	0.25	0.20
	Range	0–9.9	0–7.6	0–4.96
Mean fledge mass (g)	Mean	75.52^a^	72.47^b^	71.35^b^
	SEM	0.5	0.86	1.01
	Range	62.80–86.96	59.83–88.2	56.51–89.02

SEM, standard error of the mean.

Nest visit units are nest visits/30 min.

Brood size at day 6 included as covariate for provisioning metrics.

Different superscript letters within rows indicate means differ (*P* < 0.05) following Tukey's HSD.

**Table 2:** Annual variation in nest visit rate and reproductive success in European Starling successful peak (first) broods

	Total nest visit rate	Female nest visit rate	Male nest visit rate	Brood size fledge	Fledge mass (g)
Year	Mean ± SEM (range)	Mean ± SEM (range)	Mean ± SEM (range)	Mean ± SEM (range)	Mean ± SEM (range)
2004 *n* = 9	6.33 ± 1.35 (1–12.5)	4.28 ± 0.97 (0–9.5)	2.06 ± 0.80 (0–7.5)	3 ± 0.42 (2–5)	72.89 ± 1.55* (65.63–77.65) *n* = 8
2005 *n* = 9	7.5 ± 0.98 (3–14)	3.57 ± 0.80 (0–7)	3.93 ± 0.94 (0–9)	3.9 ± 0.29 (2–5)	76.16 ± 1.09 (70.42–80.73) *n* = 9
2010 *n* = 14	5.72 ± 0.65 (1.67–10.5)	3.65 ± 0.59 (0–6.3)	2.07 ± 0.49 (0–4.9)	3.75 ± 0.28 (2–5)	74.3 ± 1.87 (68.70–77.60) *n* = 5
2011 *n* = 10	7.05 ± 1.26 (1.5–15)	3.94 ± 0.63 (0–6)	2.71 ± 1.10 (0–9.64)	5.3 ± 0.21 (2–6)	75.77 ± 1.03 (71.20–82.00) *n* = 10
2012 *n* = 25	8.02 ± 0.68 (1–16.33)	4.81 ± 0.45 (0–10)	3.21 ± 0.47 (0–9.67)	4.52 ± 0.22 (2–6)	78.55 ± 0.80 (70.87–85.86) *n* = 26
2013 *n* = 42	6.63 ± 0.42 (2–14)	4.63 ± 0.35 (1.87–10)	2.0 ± 0.31 (0–6.43)	3.42 ± 0.16 (2–5)	74.53 ± 0.78* (62.80–86.96) *n* = 42
Total: *n* = 109	6.96 ± 0.30	4.38 ± 0.22	2.54 ± 0.23	3.92 ± 0.16	75.70 ± 0.47

^*^ Significantly lower (*P* < 0.05) than 2012.

Nest visit units are nest visits/30 min. Brood size at day 6 included as covariate for provisioning metrics. SEM= standard error of the mean.

Sample sizes for fledge mass analysis included in parentheses (total *n* = 100).

Mean fledging mass did vary among years, (*F*_5,77.1_ = 3.3, *P* = 0.01) with chicks being lightest in 2013 (post hoc Tukey's HSD *P* < 0.05, Table1).

### Repeatability of nest visit rate and relationship with individual quality metrics

**Figure 1:**
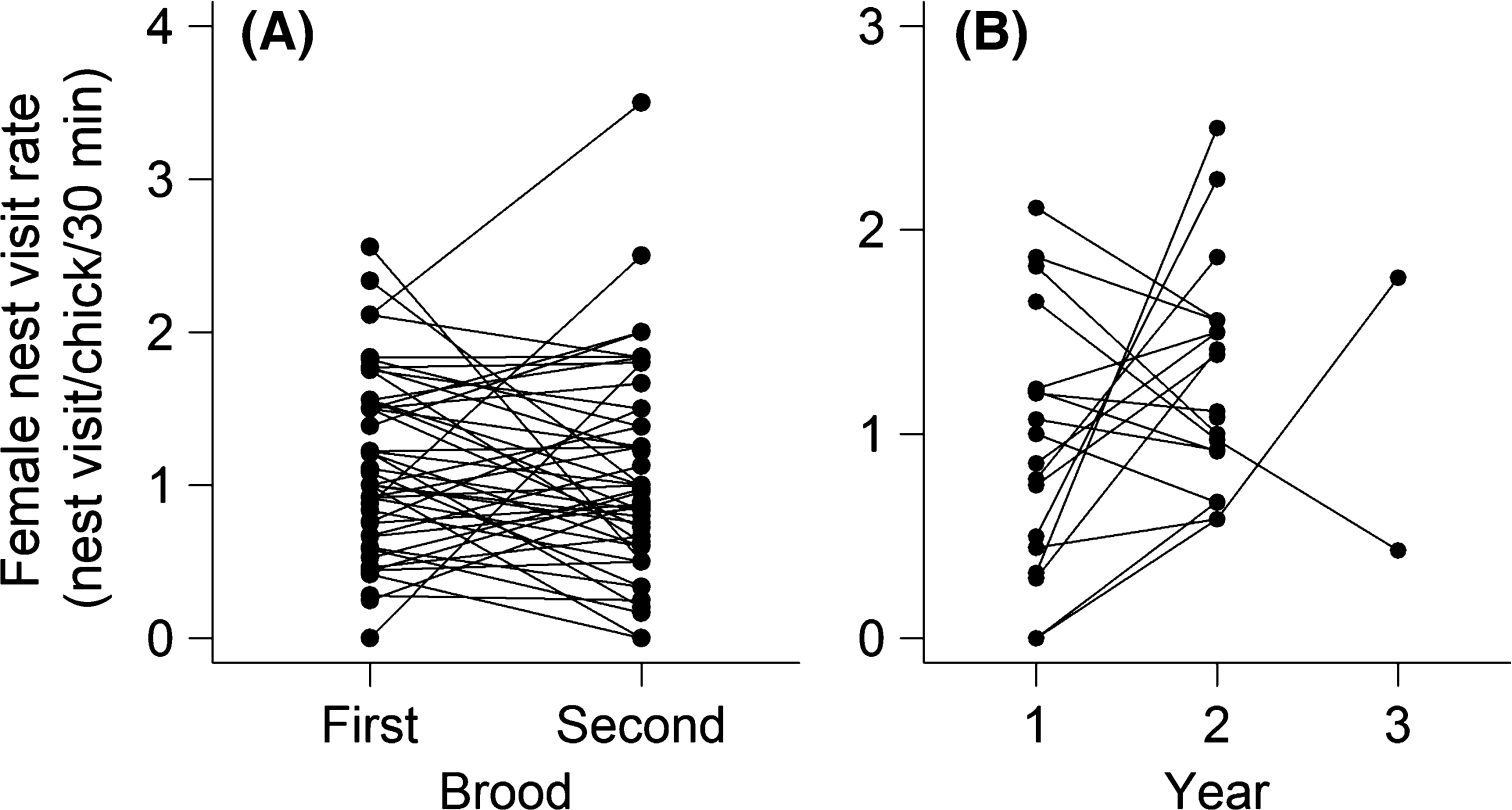
(A) Female per chick nest visit rates (nest visits/chick/30 min) for individuals who successfully reared two broods in the same year. Repeatability *r*_r_ = 0.33, *P* = 0.01. (B) Female per chick nest visit rate (nest visits/chick/30 min) for individuals who successfully reared a peak brood in more than 1 year. Repeatability *r*_r_ = 0, *P* = 0.90.

We investigated repeatability of female total nest visit rate, and per chick nest visit rate (controlling for brood size) for females who had more than one brood in a given year (*n* = 42). For these individual females, total nest visit rates were higher for peak broods (4.22 ± 0.31 visits/nest/30 min) compared to second broods (3.14 ± 0.27 visits/nest/30 min; paired *t* = 2.87, *P* = 0.006) and total nest visit rate per nest was not repeatable (repeatability calculated with LMM, *r*_r_ = 0.04, with rptR with REML calculation, *r*_r_ = 0.04, *P* = 0.34). In contrast, mean nest visit rate per chick (controlling for brood size) did not differ between peak (1.13 ± 0.09 visits/chick/30 min) and second broods (1.04 ± 0.11 visits/chick/30 min; paired *t* = 0.81, *P* > 0.05) and female nest visit rate per chick was repeatable between peak and second broods (LMM, *r* = 0.34; rptR, *r*_r_ = 0.33, *P* = 0.01; Fig.1A). We had nest visit rate data on 19 individual females that raised peak broods in two successive years, with two of those individuals breeding in 3 years. Neither female total nest visit rate (LMM, *r*_r_ = 0; rptR, *r*_r_ = 0, *P* = 0.86) nor female per chick nest visit rate (LMM, *r*_r_ = 0; rptR, *r*_r_ = 0, *P* = 0.90) was repeatable between years (Fig.1B).

**Figure 2:**
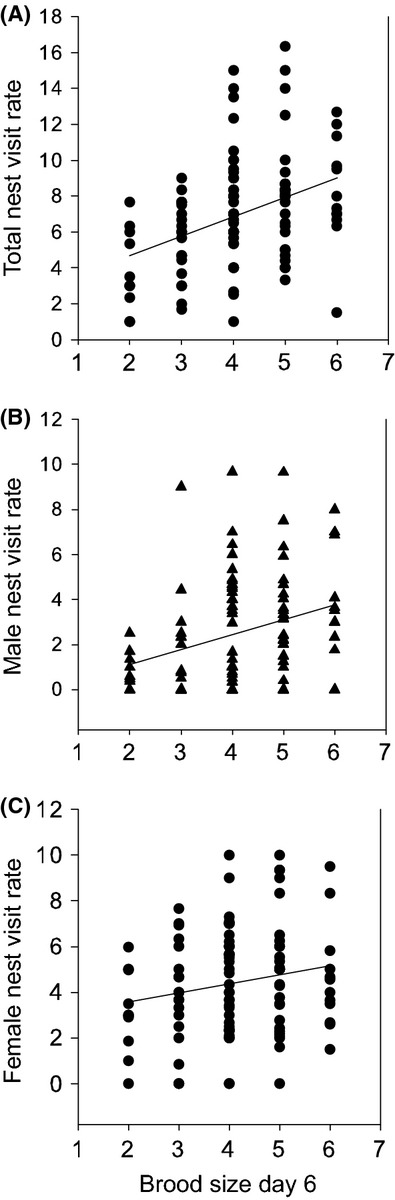
(A) Total nest visit rate (nest visits/30 min) is predicted by brood size on day 6 (*F*_1,64.3_ = 14.4, *P* = 0.003) as is (B) male only nest visit rate (*F*_1,85.7_ = 7.4, *P* = 0.008) while (C) female only nest visit rate was weakly predicted by brood size (*F*_1,79.5_ = 3.85, *P* = 0.05).

For peak broods, laying date (LD) varied by year (*F*_5,97_ = 54.9, *P* < 0.001) but neither total nest visit rate (both sexes combined) nor female nest visit rate was significantly related to LD (*P* > 0.05) (day 6 brood size included as a covariate). Female nest visit rate was not related to clutch size (*P* > 0.05), but total and male nest visit rate varied positively with clutch size (*F*_1,101.1_ = 12.4, *P* < 0.001 and *F*_1,105.4_ = 8.9, *P* = 0.004, respectively). Total nest visit rate (both sexes combined) was predicted by brood size at day 6 (*R*^2^ = 0.18, *F*_1,64.3_ = 14.4, *P* = 0.0003; Fig.2A), as was male nest visit rate (*R*^2^ = 0.08, *F*_1,85.7_ = 7.4, *P* = 0.008; Fig.2B), but female nest visit rate was only weakly, positively related to brood size at day 6 (*R*^2^ = 0.05, *F*_1,79.5_ = 3.85, *P* = 0.05; Fig.2C). Female nest visit rate was independent of the presence or absence of male help (4.2 ± 0.59 vs. 5.04 ± 0.23 visits/nest/30 min., respectively, *P* > 0.05), and there was only a weak, marginal trend for nest visit rate per chick: with male help, 1.13 ± 0.08 visits/chick/30 min., vs. no male help, 1.45 ± 0.1 visits/chick/30 min (*F*_1,104.1_ = 3.38, *P* = 0.07).

### Variation in nest visit rate and breeding productivity in peak broods

**Figure 3:**
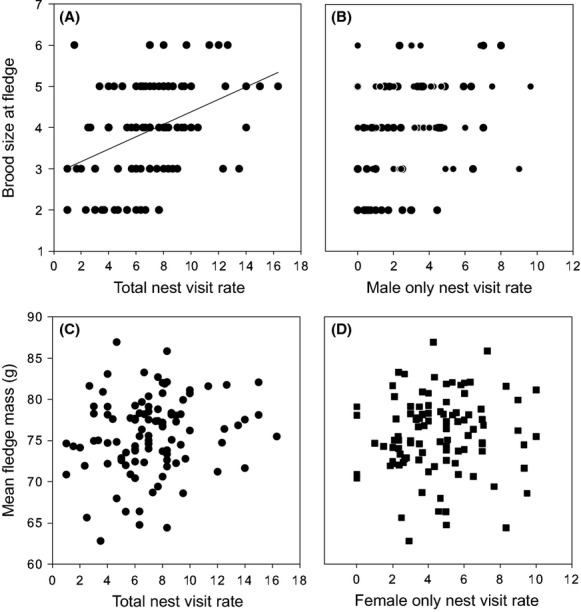
Fitness metrics relative to nest visit rate. Brood size at fledging is predicted by (A) total provisioning rate (*z* = 2.3, *P* = 0.02) and there is a nonsignificant positive trend for (B) male provisioning rate (*P* = 0.06). Mean fledge mass (g) is not predicted by (C) total nest visit rate (nest visits/30 min) or (D) female only nest visit rate (nest visits/30 min).

Brood size at fledging was positively related to total nest visit rate (both sexes combined) measured at days 6–8 posthatching (slope = 0.04, *z* = 2.3, *P* = 0.02, Fig.3A), but brood size at fledging was independent of either male or female nest visit rate when analyzed separately (*P* > 0.05; although male nest visit rate was borderline significant, *P* = 0.06, Fig.3B). Mean brood size at fledging was not different for females where males were observed contributing to provisioning (male nest visit rate > 0; mean 3.99 ± 0.13 chicks) compared with females where males were not observed (male nest visit rate = 0; mean 3.70 ± 0.25 chicks, *P* > 0.05). Similarly, mean chick mass at fledging was not different between nests with male help or without it (75.8 ± 0.57 g vs. 74.0 ± 0.99 g, *P* > 0.05). Finally, mean chick mass at fledging was not predicted by total, female or male nest visit rate (*P* > 0.05 in all cases; Fig.3C,D), and mean fledge mass did not vary with brood size (*P* > 0.05).

### Effect of experimentally delayed timing of chick-rearing on nest visit rate

Birds where laying was experimentally delayed initiated replacement clutches an average of 19 days after peak nests. We had data on experimentally delayed nests (*n* = 37) from several years (2002, 2004, 2007, 2009) and natural intermediate nests (*n* = 15) from 3 years (2007, 2010, and 2013). There were no differences between experimentally delayed and peak broods in total nest visit rate (both sexes combined) or female nest visit rate, day 6 brood size or brood size at fledge (*P* > 0.05 in all cases; Table3A). However, fledging mass was higher in peak broods than in experimentally delayed broods (*F*_1,137.8_ = 7.8, *P* = 0.006; Table3A). Fledging mass was the only significant variable (*F*_1,22.6_ = 7.6, *P* = 0.02) in the model when “date” (i.e., Julian day) was analyzed as a predictor of nest visit rates, brood sizes, and fledge mass (*R*^2^ = 0.25, Table3A), again, with chicks in peak broods fledging with higher mass than chicks in delayed broods.

**Table 3:** Provisioning behavior and reproductive success in (A) peak broods and experimentally delayed (B) and experimentally delayed and naturally intermediate broods

	Peak brood *n* = 109	Delayed *n* = 39	Delayed vs. first brood	Effect of “date”
	Mean (SEM)	Year as random	Year as random	
A				
Total nest visit rate	6.96 (0.30)	6.10 (0.41)	*F*_1,7.7_ = 3.0, *P* = 0.12	*F*_1,13.3_ = 0.52, *P* = 0.48
Female nest visit rate	4.38 (0.22)	4.05 (0.31)	*F*_1,9.7_ = 0.01, *P* = 0.93	*F*_1,12.2_ = 0.30, *P* = 0.59
Day 6 Brood Size	4.15 (0.11)	4.42 (0.17)	*z* = 0.63, *P* = 0.52	*z* = 0.71, *P* = 0.48
Brood size at fledge	3.91 (0.11)	3.85 (0.21)	*z* = −0.22, *P* = 0.82	*z* = −0.29, *P* = 0.77
Fledge mass (g)	75.52 (0.50)	72.57 (0.93)	***F***_**1,137.8**_ **= 7.8,** *P** = 0.006**	***F***_**1,22.6**_ **= 7.6,*****P*** = 0.02^*^

	Within intermediate broods		
	Experimental *n* = 39	Natural *n* = 15	
	Mean (SEM)		Mixed effects model of treatment, “natural” vs. “delay”
B			
Total nest visit rate	6.10 (0.41)	4.64 (0.77)	*F*_1,13.7_ = 3.9, *P* = 0.07
Female nest visit rate	4.05 (0.31)	3.51 (0.75)	*F*_1,10.9_ = 1.5, *P* = 0.24
day 6 Brood Size	4.42 (0.17)	3.93 (0.30)	*z* = 0.77, *P* = 0.44
Brood size at fledge	3.85 (0.21)	3.6 (0.31)	*z* = 0.42, *P* = 0.68
Fledge mass (g)	72.52 (0.93)	72.14 (2.02)	*F*_1,41.9_ = 0.0, *P* = 0.88

A: ^*^P < 0.05.Bold values highlight statistical significance.

A,B: SEM, standard error of the mean.

Nest visit units are nest visits/30 min; day 6 brood size included as covariate.

Natural intermediate broods were compared with experimentally delayed broods for the same suite of variables (mean lay date of natural intermediate broods was 5 days later than the mean lay date of experimentally delayed birds). There were no differences between naturally late laying birds and experimentally delayed birds in total, female nest visit rate, day 6 brood size, brood size at fledge, or fledge mass (*P* > 0.05, Table3B). As natural intermediate and experimentally delayed nests were not significantly different, we pooled these data to compare nest visit rate, chick number and mass between peak, intermediate, and second broods, which had mean laying dates of 30 April, 23 May, and 9 June, respectively. Brood size at fledging was different among groups (Tukey's HSD *P* < 0.05), with fewer chicks per nest in second broods than in peak and intermediate broods (Table2). Additionally, fledglings were heavier in peak compared to second and intermediate broods (*F*_2,66.9_ = 12.2, Tukey's HSD *P* < 0.001; Table2). Total nest visit rate (both sexes combined) showed a decreasing pattern across the season in intermediate and second broods (*F*_2,47.6_ = 5.7, Tukey's, HSD *P* = 0.003), but that pattern was not detected when nest visit rate was analyzed for males and females separately (*P* > 0.05; Table2).

## Discussion

In this study, we analyzed components of individual variation in work load (nest visit rate) during chick-rearing, and the consequences of this variation in terms of breeding productivity in the European starling, focusing on female birds (Williams 2012b). In this highly synchronous breeder, we predicted that the effects of date (i.e., changes in environment with time) should be less important compared to individual quality in explaining variation in provisioning effort. There was marked (10- to 16-fold) variation in total, female and male nest visit rates, among individual birds, even controlling for brood size. In females, this variation was repeatable in the short term, among consecutive days at day 6–8 posthatching, and between peak and second broods, but not among years. Individual variation in female nest visit rate was independent of environment (rainfall, temperature), other measures of individual quality (laying date and clutch size, amount of male help), and Julian date for peak broods. Furthermore, although variation in total nest visit rate (per pair) was weakly, positively correlated with chick demand, that is, brood size at days 6–8 posthatching, this was driven by the male's contribution to provisioning: the relationship of female nest visit rate to day 6 brood size was much weaker. Among broods, nest visit rate declined with date for peak, replacement, and second broods, however, brood size at fledging also decreased. Similarly, experimentally delayed birds provisioned at the same rate as peak-nesting birds supporting a lack of effect of date per se. Finally, only total nest visit rate (both sexes combined) predicted brood size at fledging and this relationship was weak, with considerable residual variation. Importantly, brood size at fledging was independent of female nest visit rate and chick mass at fledging (17 days) was independent of total, female or male nest visit rate. In other words, breeding productivity, the benefit of higher work load, was largely independent of the marked individual variation in nest visit rate, especially in females.

We predicted a priori that individual variation in nest visit rate might be affected by local weather conditions for a number of reasons, for example, low ambient temperatures might increase brood demand via an increase in chick metabolism due to thermoregulation, or it might affect prey availability and therefore foraging effort of parents (Low et al. 2008; Garcia-Navas and Sanz 2012). Numerous studies have shown that daily weather can affect chick growth, although mainly at extremes of temperature and in younger chicks (Keller and Van Noordwijk 1994; Cunningham et al. 2013; Winkler et al. 2013), but these studies often do not also consider variation in nest visit rate. Daily feeding rates per chick have been reported to be negatively (*Cyanistes caeruleus*), (Garcia-Navas and Sanz 2012) or positively (Low et al. 2008) related to temperature, or to be independent of daily temperature (Barba et al. 2009). We did not detect any relationships between nest visit rate and environmental variables (rain or temperature) during the period when we measured provisioning effort, perhaps because the high temporal synchrony of peak broods in European starlings means that most parents encounter the same environmental variables (food availability, inclement weather, etc.) during the relatively short phase of chick rearing. Thus, environmental factors on short temporal scales contributed relatively little to the marked individual variation in parental nest visit rate that we documented.

We found that total nest visit rate, of both parents, was related to current chick demand, defined here as brood size at day 6 (as in (Bortolotti et al. 2011; Garcia-Navas and Sanz 2011), although this was mostly driven by males (*R*^2^ = 0.08) and there was still substantial residual variation (*R*^2^ total nest visit rate = 0.18; see Fig.2B). However, we found no evidence that variation in female nest visit was related to other measures of female quality, including laying date and clutch size. Furthermore, we could detect no (or only a marginal) difference in nest visit rate for females where males were observed contributing to provisioning of offspring (likely higher quality or “primary” females, Sandell et al. 1996) compared with females where males were not observed (likely “secondary” females). Nevertheless, individual variation in female per chick nest visit rate was repeatable in the short term between peak and 2nd broods, despite average brood sizes being smaller in second broods, which also supports the idea that females do not adjust their provisioning effort to brood size. Numerous studies have reported significant repeatability of provisioning effort within years, although typically male effort is repeatable while female provisioning effort is less repeatable or not repeatable (Freeman-Gallant and Rothstein 1999; Maccoll and Hatchwell 2003; Schwagmeyer and Mock 2003; Cleasby et al. 2013). However, results are mixed even for the same species perhaps suggesting strong context-dependence for this relationship, for example, in house sparrows, only males (Schwagmeyer and Mock 2003; Nakagawa et al. 2007; Cleasby et al. 2013), or both males and females (Dor and Lotem 2010) showed within year repeatability. There is much more limited evidence that repeatability reflects heritable variation in nest visit rates (Dor and Lotem 2010 but see Maccoll and Hatchwell 2003), especially in females (Freeman-Gallant and Rothstein 1999; Gray et al. 2005; Nakagawa et al. 2007), which is consistent with our result of lack of repeatability of provisioning effort across years in female European starlings.

We found no effect of laying date (time) on variation in nest visit rate for peak broods, which is not surprising given the high level of breeding synchrony in our population of European starlings (Williams et al. 2015). Nest visit rate did decline with date between peak, replacement, and second broods; however, brood size at fledging also decreased so provisioning effort per chick remained constant. Similarly, experimentally delayed birds provisioned at the same rate as peak-nesting birds (which suggests that the cost of producing a second clutch of eggs due to our egg removal, was not sufficient to generate immediate negative consequences). These results support a lack of effect of date per se*,* but they are consistent with idea that individual females are committed to a certain level of parental care at the outset of the breeding season that is maintained across breeding attempts within a year but not among years (see below). In tree swallows (*Tachycineta bicolor*), similarly delayed hatching did not reduce nest visit rate despite a seasonal decline in food abundance (Bortolotti et al. 2011). In contrast, in blue tits early breeding pairs that were experimentally delayed exhibited higher nest visit rates and raised significantly heavier nestlings than expected which suggests that birds attempted to compensate for less favorable environmental conditions later in the breeding season (Garcia-Navas and Sanz 2011).

Despite the marked (10-fold) variation in parental effort exhibited by European starlings, as measured by nest visit rate, we found little evidence that parents, especially females, with high nest visit rates benefited from this higher workload in terms of the number or quality of chicks fledged. In our study, brood size at fledging was predicted by total nest visit rate, but this effect was mostly driven by males, as male nest visit rate alone was borderline significant, and this relationship was weak with >fivefold variation in nest visit rate for any given brood size (see Fig.3A,B). Brood size at fledging was independent of female nest visit rate and, in addition, we could detect no difference in productivity for females where males were observed contributing to provisioning of offspring (likely higher quality or “primary” females, Sandell et al. 1996; see above) and those where males were not observed helping – again suggesting an uncoupling of workload as measured by nest visit rate and productivity. Furthermore, chick mass at fledging (17 days of age) was independent of any measure of nest visit rate. Surprisingly, although some studies have investigated relationships between brood size and provisioning effort in young, prefledged chicks, they have less often considered measures of breeding productivity at fledging (e.g., Bortolotti et al. 2011; Garcia-Navas and Sanz 2011). Although it is widely assumed that parental workload, most commonly measured as nest visit rate, should be positively related to breeding productivity given the predicted high costs of flight and foraging effort (e.g., (Tinbergen 1981; Ward et al. 2001), numerous studies have failed to find this relationship (Rytkönen et al. 1995; Schwagmeyer and Mock 2008; Ringsby et al. 2009; Mariette et al. 2011; Garcia-Navas and Sanz 2012). Furthermore, in several species fledging mass was predicted by male provisioning rate, but was unrelated to variation in female nest visit rate (Magi et al. 2009; Stodola et al. 2010). In studies where female feeding visits are correlated with nestling growth rate this relationship is weak (e.g., *r*^2^ = 0.18, Maigret and Murphy 1997; Ardia 2007), that is, most of the individual variation in female parental effort remains unexplained (see also Shutler et al. 2006). Of course, one potential explanation for the lack of relationship between costs of parental effort and benefits in terms of number or quality of chicks is that nest visit rate (the most widely used metric of parental effort) does not accurately measure workload. Birds could vary workload through variation in meal size, foraging distance, or the size or quality of prey (Wright et al. 1998; Stodola et al. 2010), for example, fledging mass can be predicted by delivery rate of the largest (Schwagmeyer and Mock 2008) or rarest prey items (Wright et al. 1998). Clearly, it will be important in future studies to quantify meal size, prey quality, foraging distance, etc. (Williams and Fowler 2015), although this is technically challenging for large samples of individuals (such as we analyze here) and there is some evidence that this will not provide a simple explanation for the dissociation between parental effort and productivity. Some studies have confirmed that nest visit rate is an accurate measure of food delivery (McCarty 2002) and even studies using alternate metrics of parental workload, for example, estimated prey biomass delivered to nestlings, or flight distance while foraging, have failed to find relationships with fledging mass or chicks number (Strauss et al. 2005; Stodola et al. 2010; Garcia-Navas and Sanz 2011). Furthermore, experimental manipulation of parental effort during chick-rearing in small passerines (e.g., using clipping of flight or tail feathers, or addition of small weights), which generally reduce nest visit rates in manipulated bird, has relatively little, or no, effect on fledging success, chick growth, or fledging mass (Verbeek and Morgan 1980; Slagsvold and Lifjeld 1988; Wright and Cuthill 1989; Winkler and Allen 1995; Love and Williams 2008; but see Slagsvold and Lifjeld 1990; Moreno et al. 1999). The fact that variation in male nest visit rate does show some systematic patterns (e.g., repeatability) suggests that the dissociation between individual variation in female provisioning and productivity might be a biological difference not a methodological artifact (Williams and Fowler 2015).

We interpret our results as supporting the idea that the level of provisioning behavior in individual female European starlings is fixed within a season, over multiple breeding attempts, presumably based on assimilated (albeit currently unknown) cues but which is independent of their mates' behavior. Schwagmeyer et al. (2002) also suggested that variation in nestling provisioning in house sparrows (*Passer domesticus*) was largely attributable to factors that were independent of the mate's current behavior, and they suggested this reflected differences in individual quality of females. In the same species, Westneat et al. (2011) suggested that provisioning rate is influenced by both personality and plasticity, and that males and females are influenced by different variables (although a large amount of residual variation remained unexplained in their analysis). Our data also suggest the marked individual variation in female provisioning rates reflects individual plasticity among years, which would be consistent with an individuals' ability to adjust to year-specific environmental conditions, for example, food availability or mate quality. This individual plasticity might be related to some measure of phenotypic quality that we did not measure, but it was independent of early season, fecundity components of quality: laying date or clutch size in our study (cf. Schwagmeyer et al. 2002).

In summary, the current lack of identifiable causes and consequences of the marked variation in provisioning rate suggests a need for re-evaluating the framework for predictability and plasticity of parental investment, especially at the individual level and especially in females (Nakagawa et al. 2007; Williams 2012b; Williams and Fowler 2015). Our data support the hypothesis that individual females reassess their environment each year and determine their seasonally fixed workload, that is, there is a consistency of individual behavior across breeding attempts within a year, which is largely independent of time (date), brood demand or mate quality. Seasonally fixed, but annually variably behavior is consistent with the idea that individuals behave as if they had committed to a certain level of parental care at the outset of their annual breeding attempt(s). Our data suggest that, at least in European starlings, individual variation in parental care (i.e., secondary reproductive effort) does not reflect, or can be uncoupled from, metrics of individual quality for primary reproductive effort (timing of laying, fecundity). Nakagawa et al. (2007) suggested that there are “predictable males [but] unpredictable females”. We would argue that individual females are in fact making predictable decisions about their workload during provisioning that maximizes their overall fitness (i.e., individual optimization) based on an integration of current large scale environmental (e.g., food availability, Low et al. 2012) or social cues. If we can better identify these cues, and the physiological mechanisms that mediate these cues (similar to the physiological response mechanisms for timing of breeding decisions sensu Visser et al. 2010), then the predictable nature of individual variation in female provisioning behavior will likely become apparent.

## Appendix

**A1:** Summary of models run. Unless noted, linear mixed effects models were run with the lme4 package in R

Response variable	Fixed effects	Random effects	*P* value	Notes
Environmental effects				
Total nest visit rate	Rain, temperature, BS6	Female ID, Year	NS	
Female nest visit rate	Rain, temperature, BS6	Female ID, Year	NS	
Male nest visit rate	Rain, temperature, BS6	Female ID, Year	NS	
Differences between years (peak nests)				
Total nest visit rate	Year, BS6	Female ID	NS	
Female nest visit rate	Year, BS6	Female ID	NS	
Male nest visit rate	Year, BS6	Female ID	NS	
BSF	Year	Female ID	NS	GLMM with poisson distribution
Fledge mass	Year	Female ID	0.01	
Lay Date	Year	Female ID	<0.001	
Clutch size and laydate relative to nest visit rate (peak broods)				
Total nest visit rate	Clutch size	Female ID, Year	<0.001	
Female nest visit rate	Clutch size	Female ID, Year	NS	
Male nest visit rate	Clutch size	Female ID, Year	0.004	
Total nest visit rate	Lay Date, BS6	Female ID, Year	NS	
Female nest visit rate	Lay Date, BS6	Female ID, Year	NS	
Male nest visit rate	Lay Date, BS6	Female ID, Year	NS	
Peak brood nest visit variation				
Total nest visit rate	BS6	Female ID, Year	0.0003	
Female nest visit rate	BS6	Female ID, Year	0.05	
Male nest visit rate	BS6	Female ID, Year	0.008	
Female nest visit rate	Male help (yes or no)	Female ID, Year	NS	
Fitness metrics (peak broods)				
BSF	Total nest visit rate	Female ID, Year	0.02	GLMM with poisson distribution
BSF	Female nest visit rate	Female ID, Year	NS	GLMM with poisson distribution
BSF	Male nest visit rate	Female ID, Year	NS (0.06)	GLMM with poisson distribution
BSF	Male help (yes or no)	Female ID, Year	NS	GLMM with poisson distribution
Fledge mass	Total nest visit rate,	Female ID, Year	NS	
Fledge mass	Female nest visit rate	Female ID, Year	NS	
Fledge mass	Male nest visit rate	Female ID, Year	NS	
Fledge mass	Male help (yes or no)	Female ID, Year	NS	
Differences between broods				
Total nest visit rate	Brood, BS6	Female ID, Year	0.006	
Female nest visit rate	Brood, BS6	Female ID, Year	NS	
Male nest visit rate	Brood, BS6	Female ID, Year	NS	
BSF	Brood	Female ID, Year	<0.001	GLMM with poisson distribution
Fledge mass	Brood	Female ID, Year	<0.001	
Experimentally delayed and peak broods				
Total nest visit rate	Treatment, BS6	Female ID, Year	NS	
Female nest visit rate	Treatment, BS6	Female ID, Year	NS	
BS6	Treatment	Female ID, Year	NS	GLMM with poisson distribution
BSF	Treatment	Female ID, Year	NS	GLMM with poisson distribution
Fledge mass	Treatment	Female ID, Year	0.006	
Total nest visit rate	Date, BS6	Female ID, Year	NS	
Female nest visit rate	Date, BS6	Female ID, Year	NS	
BS6	Date	Female ID, Year	NS	GLMM with poisson distribution
BSF	Date	Female ID, Year	NS	GLMM with Poisson distribution
Fledge mass	Date	Female ID, Year	0.01	
Naturally intermediate and experimentally delayed broods				
Total nest visit rate	Treatment, BS6	Female ID, Year	NS	
Female nest visit rate	Treatment, BS6	Female ID, Year	NS	
BS6	Treatment	Female ID, Year	NS	GLMM with poisson distribution
BSF	Treatment	Female ID, Year	NS	GLMM with poisson distribution
Fledge mass	Treatment	Female ID	NS	Won't converge with Year as random

BS6: Brood size at day 6.

BSF: Brood size at fledge.

NS: not significant, *P* > 0.05.

Nest visit rate: visits/30 min.
